# Supplementary material for: Chip collection of hepatocellular carcinoma based on O2 heterogeneity from patient tissue
Source: Nat Commun. 2024 Jun 15;15:5117. doi: 10.1038/s41467-024-49386-8 (PMC11180182; doi:10.1038/s41467-024-49386-8)
Supplement: Supplementary file 1 — Supplementary information [file 41467_2024_49386_MOESM1_ESM.pdf]

Supplementary information

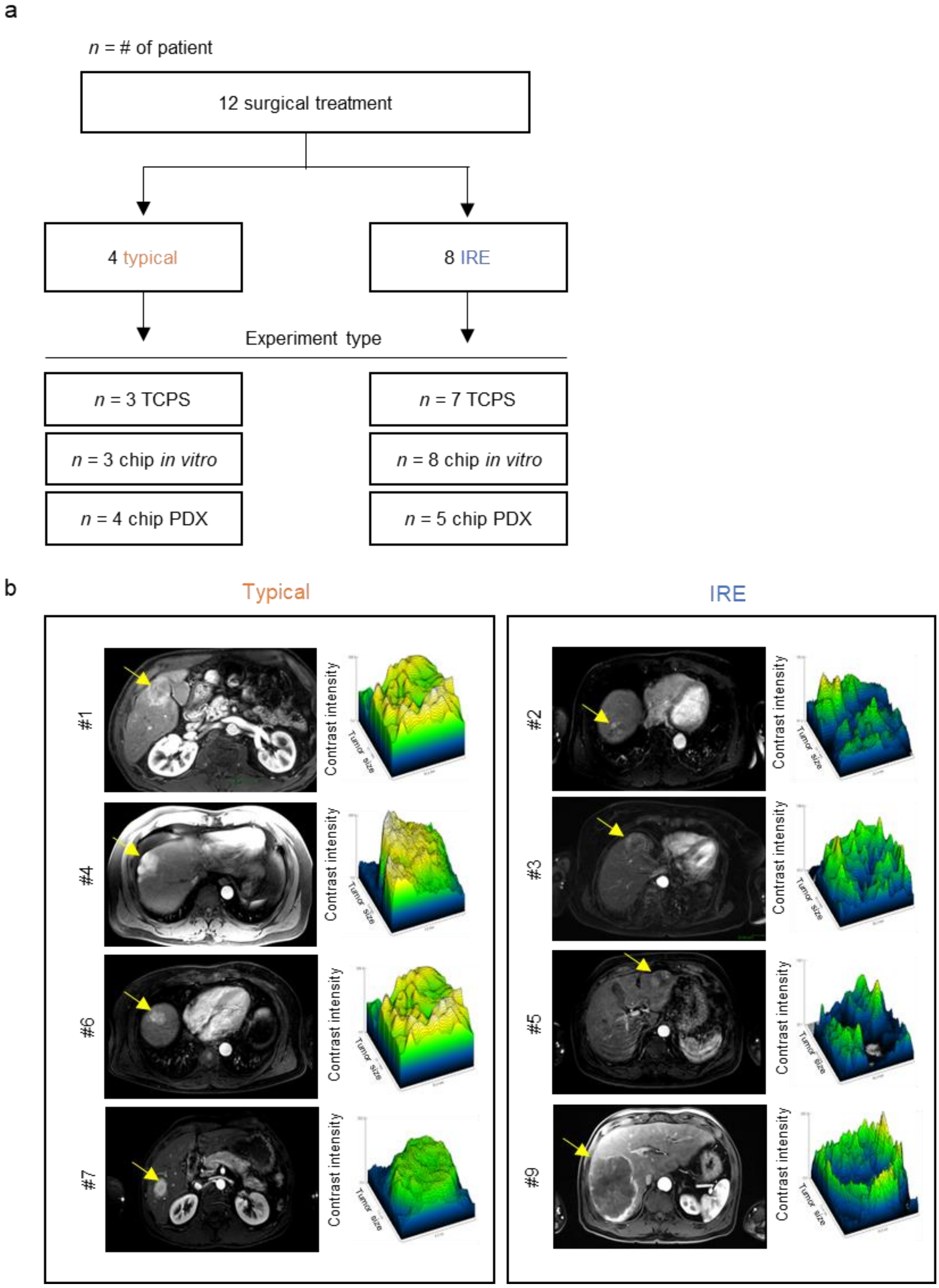

Supplementary Fig. 1 | Classification of twelve patients with HCC based on cell type. a. HCC tissue samples were obtained from 12 patients during surgery for the experiments. Among them, 4 samples of typical HCC tissues were used for TCPS (*n* = 3), *in vitro* chip (*n* = 3), and chip PDX (*n* = 4) experiments. Additionally, 8 samples of IRE tissues, collected from patients with a median follow-up of 19.8 months, were used for TCPS (*n* = 7), *in vitro* chip (*n*

= 8), and chip PDX (*n* = 5) experiments. **b.** Segmentation of the MRI contrast signal (yellow arrow) for 3D intensity projection reveals reduced contrast intensity at the core of IRE HCCs, accompanied by rim-like enhanced intensity along the tumor border. Conversely, typical HCCs exhibit overall enhanced intensity within the tumor, characterized by a border peak pattern.

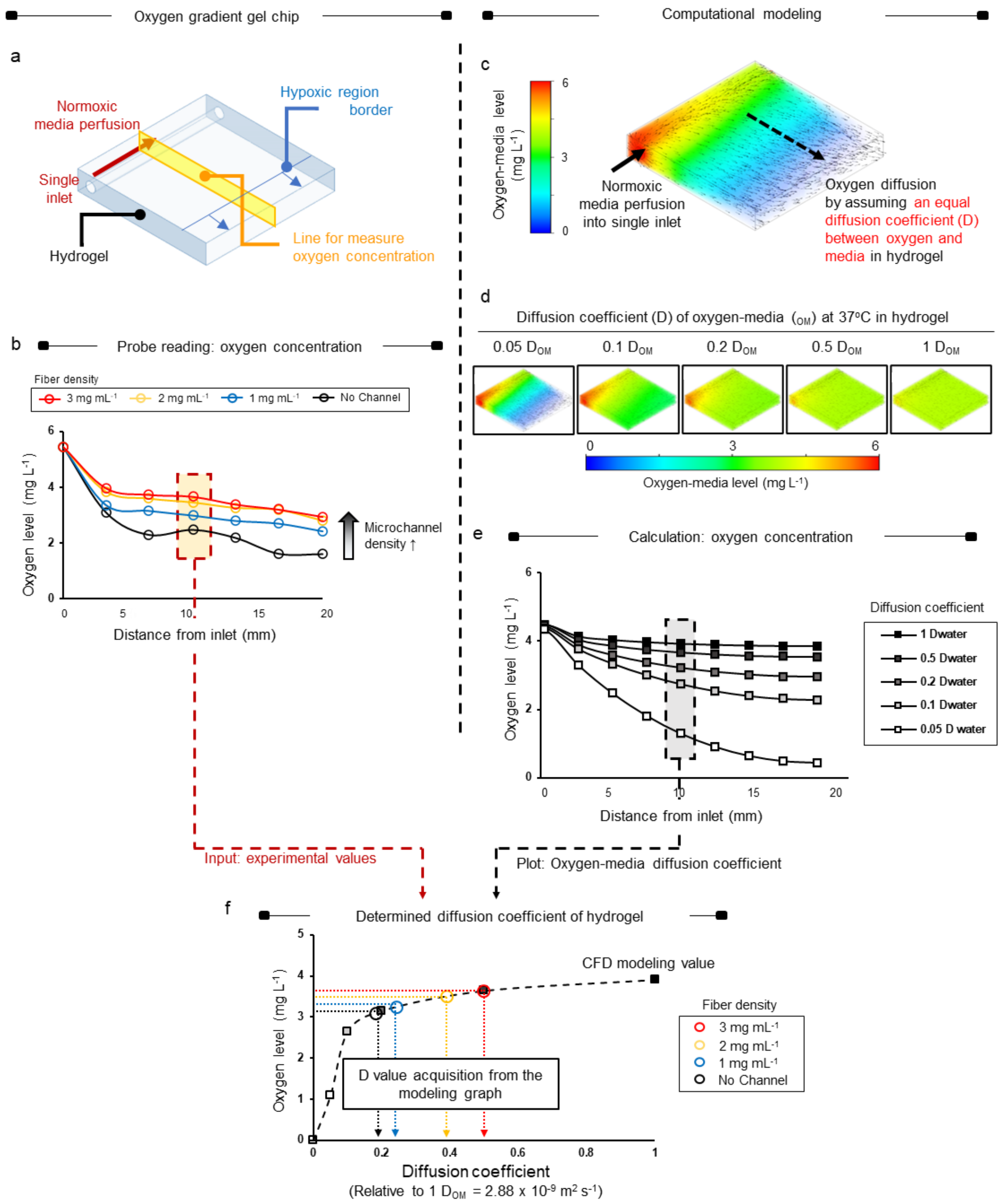

Supplementary Fig. 2 | Acquisition of diffusion coefficient as a function of fiber density. **a.** A single inlet chip (red) is perfused with normoxic media containing an oxygen concentration of 5.57 mL^{-1}. This configuration is designed to maximize the effects of channel density, a crucial factor governing oxygen media perfusion through the chip hydrogel. An oxygen gradient is generated within the hydrogel (sky blue) with the hypoxic region delineated by the border (blue). Using a probe, oxygen levels are measured after 1 minute of

media perfusion along the line (yellow) at incremental distances from the inlet. **b**. An increase in microchannel density (0, 1, 2, 3 mg mL^{-1}) correlates with increased overall oxygen diffusion at each measurement point. The linearity of the decremental slope also improves with increasing distance from the inlet. **c.** Assuming an equal diffusion coefficient between the media and oxygen during perfusion, the CFD modeling of the gradient projects a color-contour variation throughout the hydrogel in the chip. **d.** The diffusion coefficient (D) of oxygen-carrying media (water) at 37°C is defined as D_{OM}. It decreases proportionally as the D_{OM} ratio is reduced from 1 (1 $D_{OM} = 2.88 \times 10^{-9}$ m^2 s^{-1}) to 0.5, 0.2, 0.1, and further to 0.05. Subsequently, alterations in color contour indicate changes in the gradient pattern, with the most pronounced decrease in oxygen levels observed at 0.05 D_{OM} along the incremental distance from the inlet. **e.** The CFD results allow for the calculation of oxygen levels at incremental distances from the inlet as a function of the D_{OM} ratio. These findings confirm the most pronounced decrease in oxygen levels observed at 0.05 D_{water}, with the lowest level recorded at the measurement point. **f.** The oxygen level (y-axis, mgL^{-1}) is plotted as a function of the D_{OM} ratio (x-axis, relative to 1 $D_{OM} = 2.88 \times 10^{-9}$ m^2 s^{-1}) based on CFD calculation. Next, the oxygen levels recorded by the probe are mapped onto the y-axis to derive the actual D values as a function of fiber density in the chip hydrogel. As the fiber density increases, there is a corresponding increase in the D value. Source data are provided as a Source Data file.

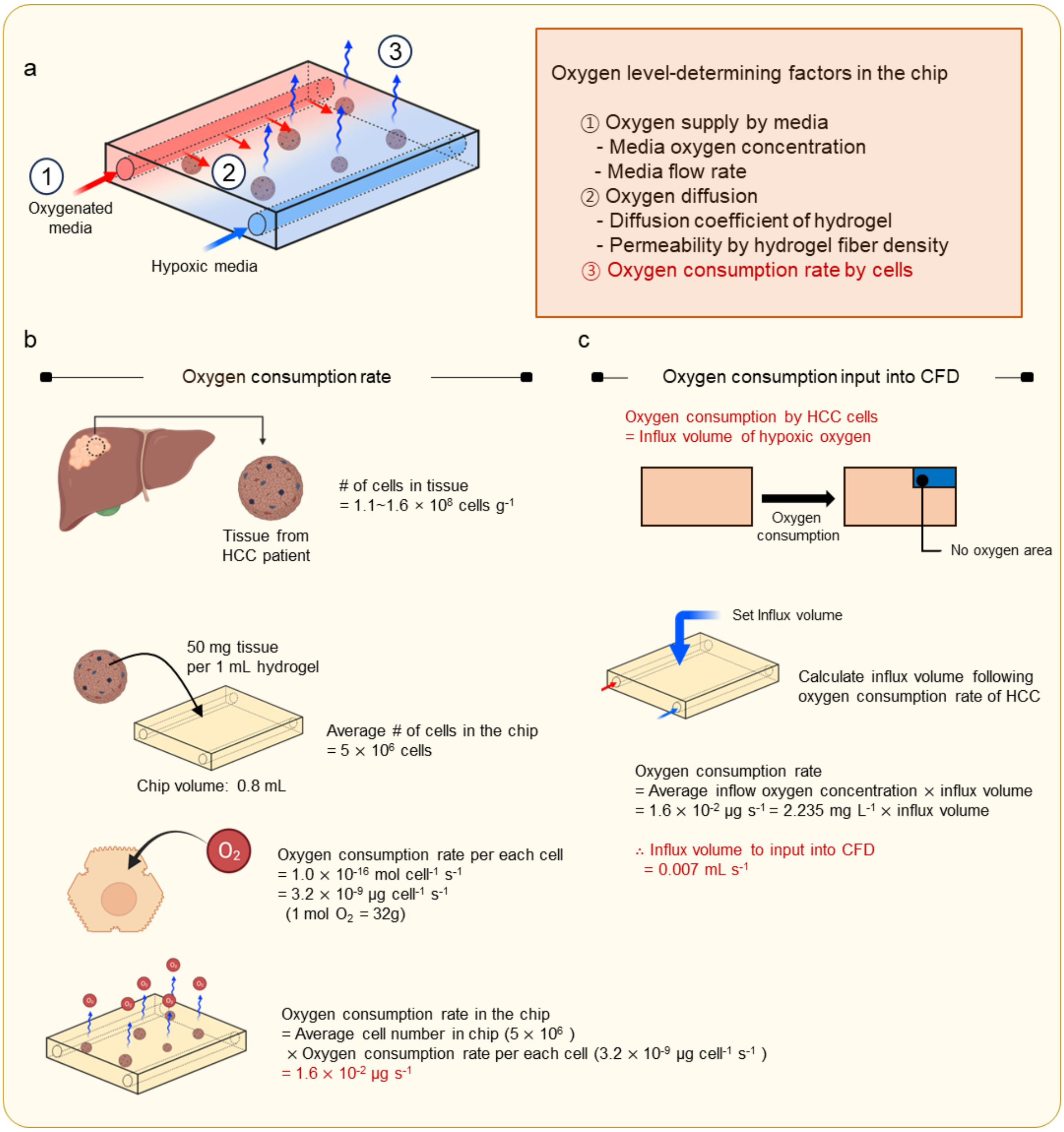

Supplementary Fig. 3 | Determining hypoxic oxygen influx volume for CFD modeling using oxygen consumption rates. a. The oxygen gradient within the chip is established by regulating boundary conditions such as media supply factors (oxygen concentration and flow rate) and diffusion parameters (D and permeability of hydrogel). Moreover, the oxygen consumption rate of HCC cells plays a crucial role in establishing the gradient through CFD modeling. **b.** The oxygen consumption rate in the chip is determined to be 1.6×10^{-2} μg s^{-1} through a series of calculations involving the number of cells in the HCC tissue, the average number of cells in the chip, and then, the O_2 consumption rate per each cell. **c.** The influx

volume of hypoxic oxygen is calculated (0.007 mL s^{-1}) as an input value for the oxygen consumption by HCC cells to be used in CFD modeling.

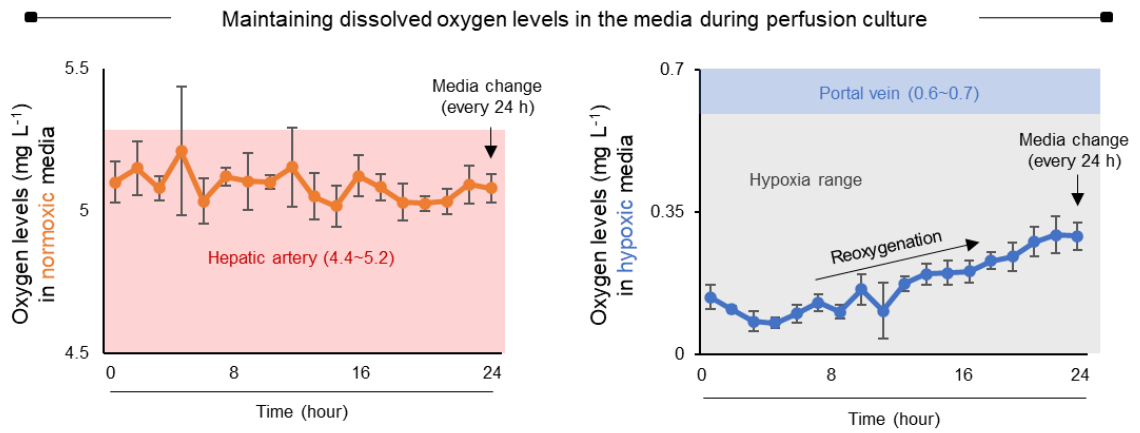

Supplementary Fig. 4 | Monitoring oxygen level changes in the medium during perfusion culture. Dissolved oxygen levels are monitored continuously for 24 hours in the normoxic and hypoxic media under perfusion into the oxygen gradient chip. Minimal fluctuations are detected in the normoxic media. The hypoxic medium consistently maintains the oxygen concentration below 0.35 mg L^{-1}, which is lower than the physiological range in the portal vein (0.6~0.7 mg L^{-1}). However, there is a gradual increase in the overall trend of oxygen level, suggesting the need of media exchange every 24 hours. Data are shown as mean ± SD, n = 3 biological replicates. Source data are provided as a Source Data file.

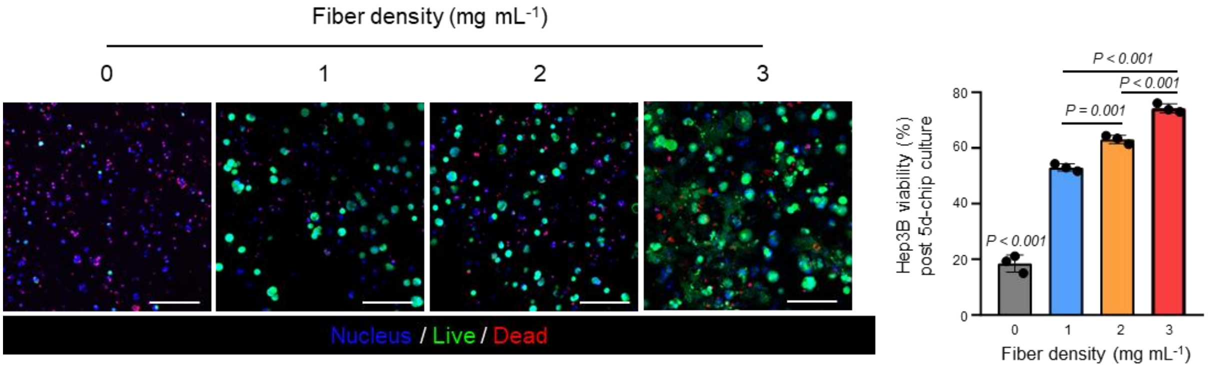

Supplementary Fig. 5 | Improvement of cell viability by increasing the fiber density. The viability of Hep3B cells is improved upon live and dead assays as the fiber density increases during the 5-day perfusion culture (0 to 3 mg mL^{-1}, Scale bars = 200 μm). Data = mean ± SD, *n* = 3 biological replicates. Significance was determined using one-way ANOVA with Tukey's test versus between lined groups. Source data are provided as a Source Data file.

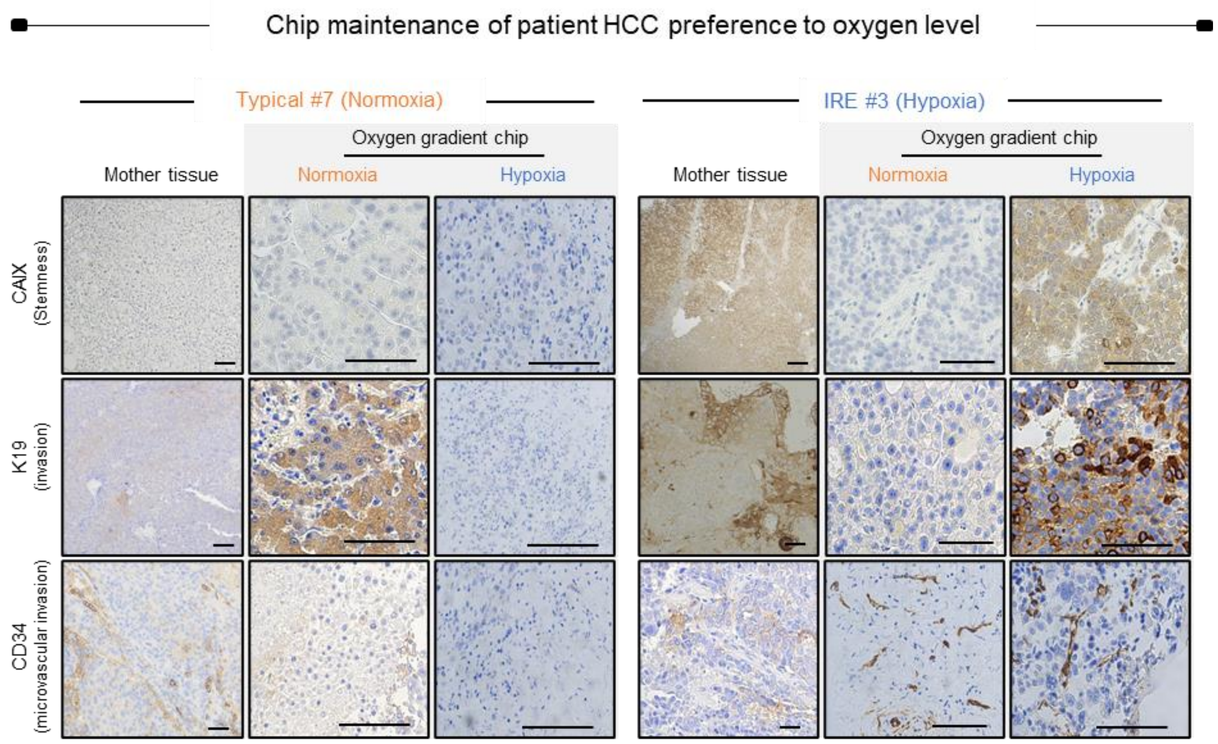

Supplementary Fig. 6 | Discerning HCC type-dependent preference of oxygen level by the chip. When HCC patient tissues are cultured on the chip for 4 days, typical (#7) and IRE (#3) HCCs show increased expression of protein markers associated with hypoxia-mediated stemness (CAIX), cancer invasiveness (K19), and microvascular invasion (CD34) on the normoxic and hypoxic sides, resecptively, compared to the corresponding mother tissue and the opposite side. The results confirmed that the chip effectively preserves the inherent tissue characteristics related to oxygen preference (Scale bar = 200 μm).

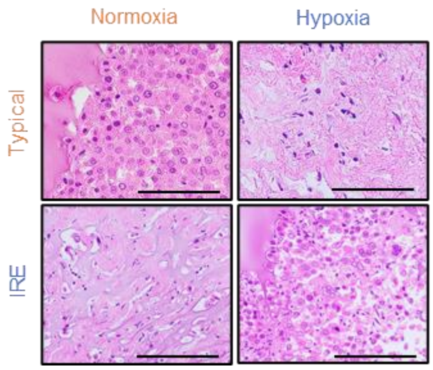

Supplementary Fig. 7 | Preference of HCC patient tissues to oxygen levels of the gradient chip. During the 4-day culture of HCC tissues in the oxygen gradient chip, the IRE type exhibits abundant nuclei in hypoxic conditions, indicating rapid HCC propagation compared to the sparse nuclei observed in normoxic conditions. This pattern contrasts with the typical type, as revealed by H&E staining (Scale bar = 200 μm).

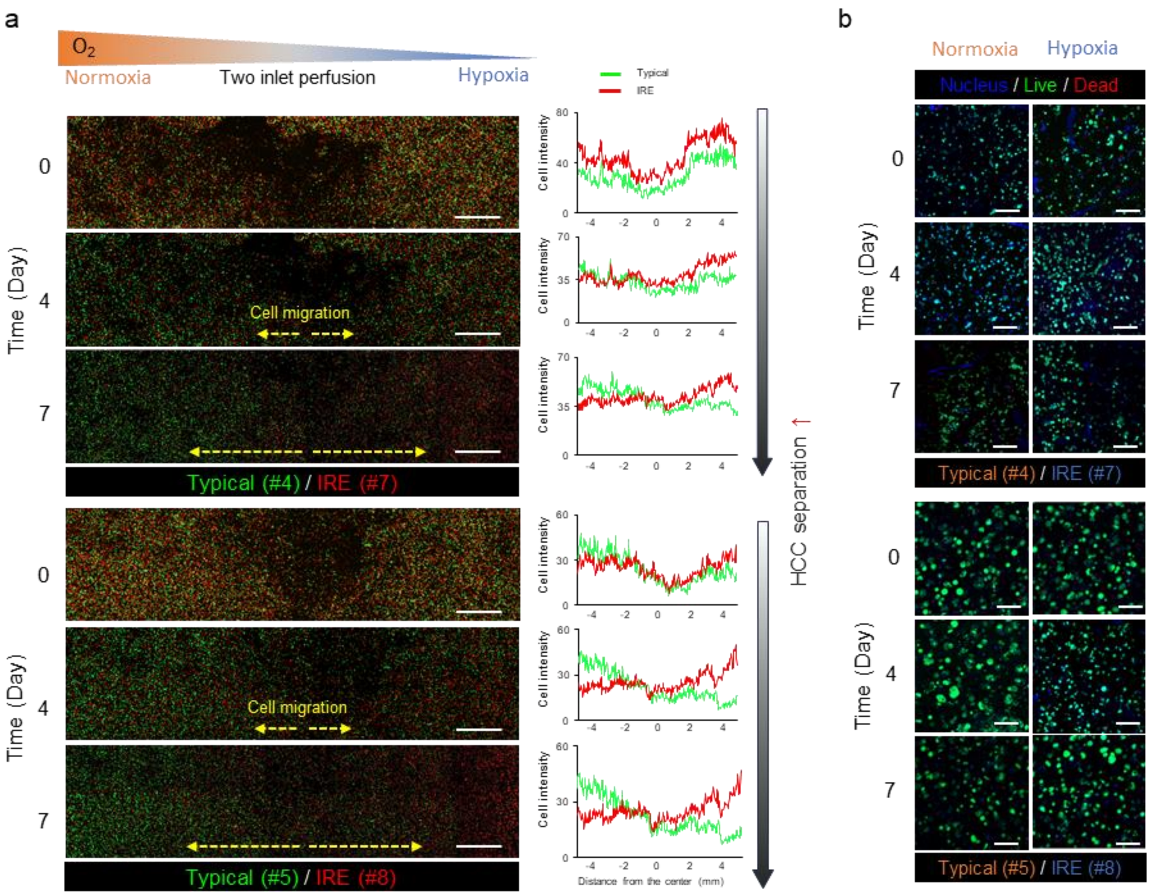

Supplementary Fig. 8 | On-chip separation and collection of viable HCC cells based on oxygen levels. a. A mixture of typical (T) and IRE (I) HCC cells (Top: T#4+I#7 or Bottom: T#5+I#8) is cultured on the chip for 7 days. Confocal imaging shows that over time, IRE cells (red DiI) progressively migrate to the hypoxic side (to the right), while the typical cells (green DiO) remain predominantly on the normoxic side (to the left). After the 7-day culture period, the two types of HCC cells are separated and collected on the normoxic and hypoxic sides, respectively, as shown in the day-7 histogram (n = 3 biological replications, Scale bar = 1 mm). **b.** During the 7-day collection period in the chip, the HCC cells remain viable without experiencing significant cell death (n = 3 biological replications, Scale bar = 100 µm). Source data are provided as a Source Data file.

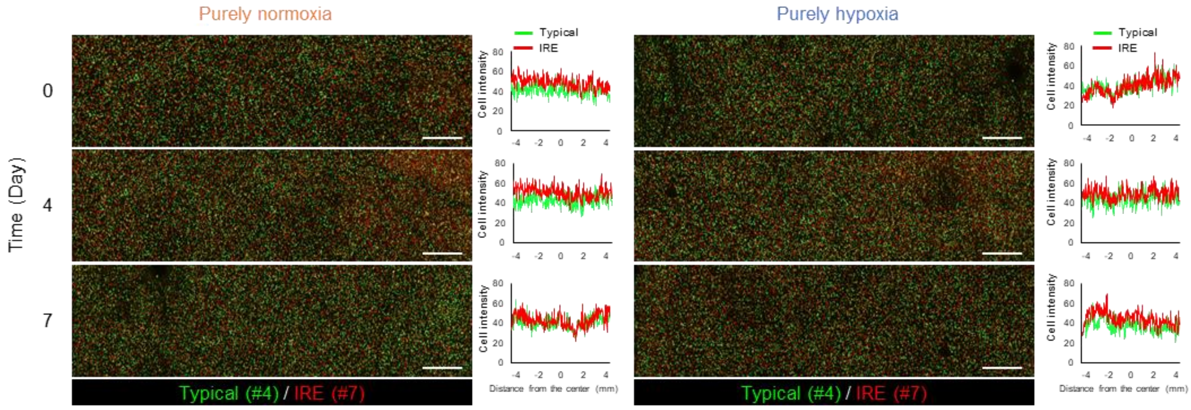

Supplementary Fig. 9 | No separation of HCC cells by uniform oxygen environments. A mixture of typical HCC cells (#4, green) and IRE HCC cells (#7, red) is cultured under uniform normoxia or uniform hypoxia for 7 days, resulting in no clear separation under confocal imaging. The result is validated by histograms for days 0, 4, and 7. (*n* = 3 biological replications, Scale bar = 1 mm). Source data are provided as a Source Data file.

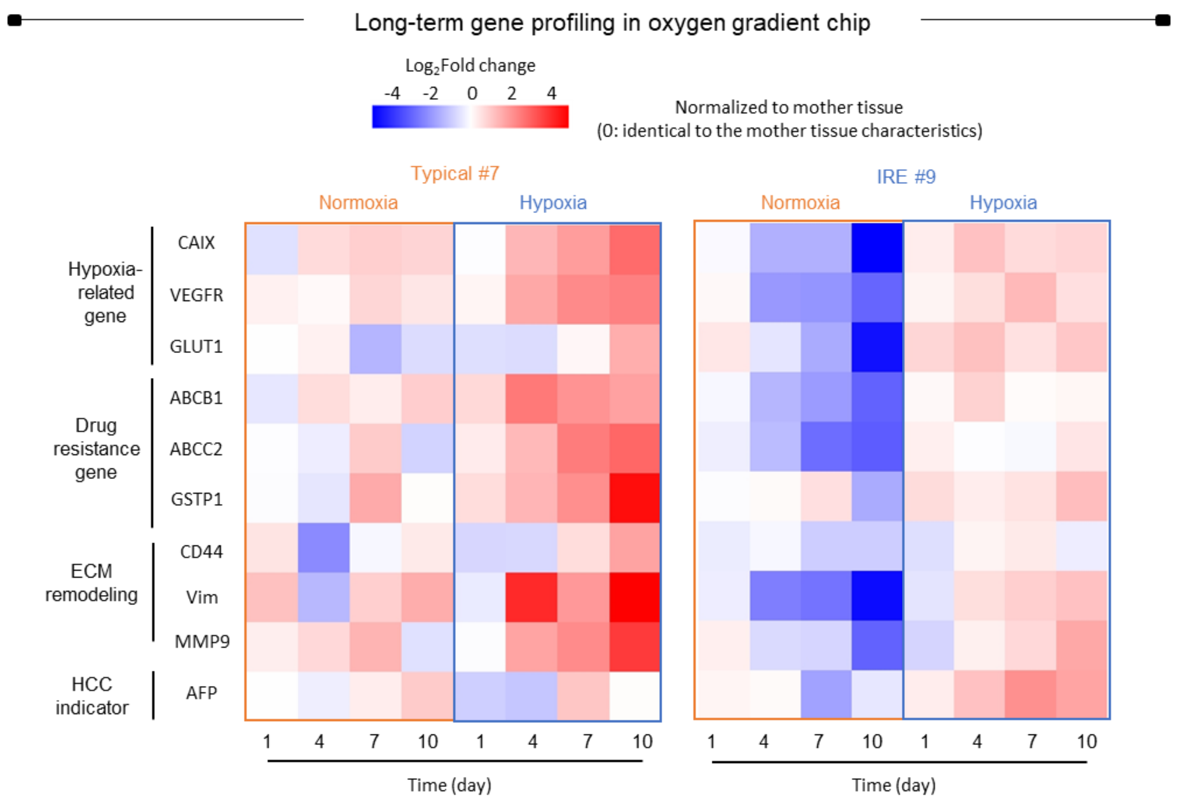

Supplementary Fig. 10 | 10-day maintenance of phenotypic gene profiles in patient cells upon chip culture. In the normoxic zone, typical (#7) cells retain the gene expression profile of mother tissue (0: white) with respect to hypoxia (*CAIX*, *VEGF*, *GLUT1*), drug resistance (*ABCB1*, *ABCC2*, *GSTP1*), and ECM remodeling (*CD44*, *Vim*, *MMP9*) and HCC indicator (*AFP*) for 10 days in the chip culture. In contrast, the hypoxia specific-maintenance (0: white) of mother tissue characteristic is dominantly seen in IRE (#9) cells. The results are presented as Log_2Fold change values with normalization to those of corresponding mother tissue (n = 3 biological replications). Source data are provided as a Source Data file.

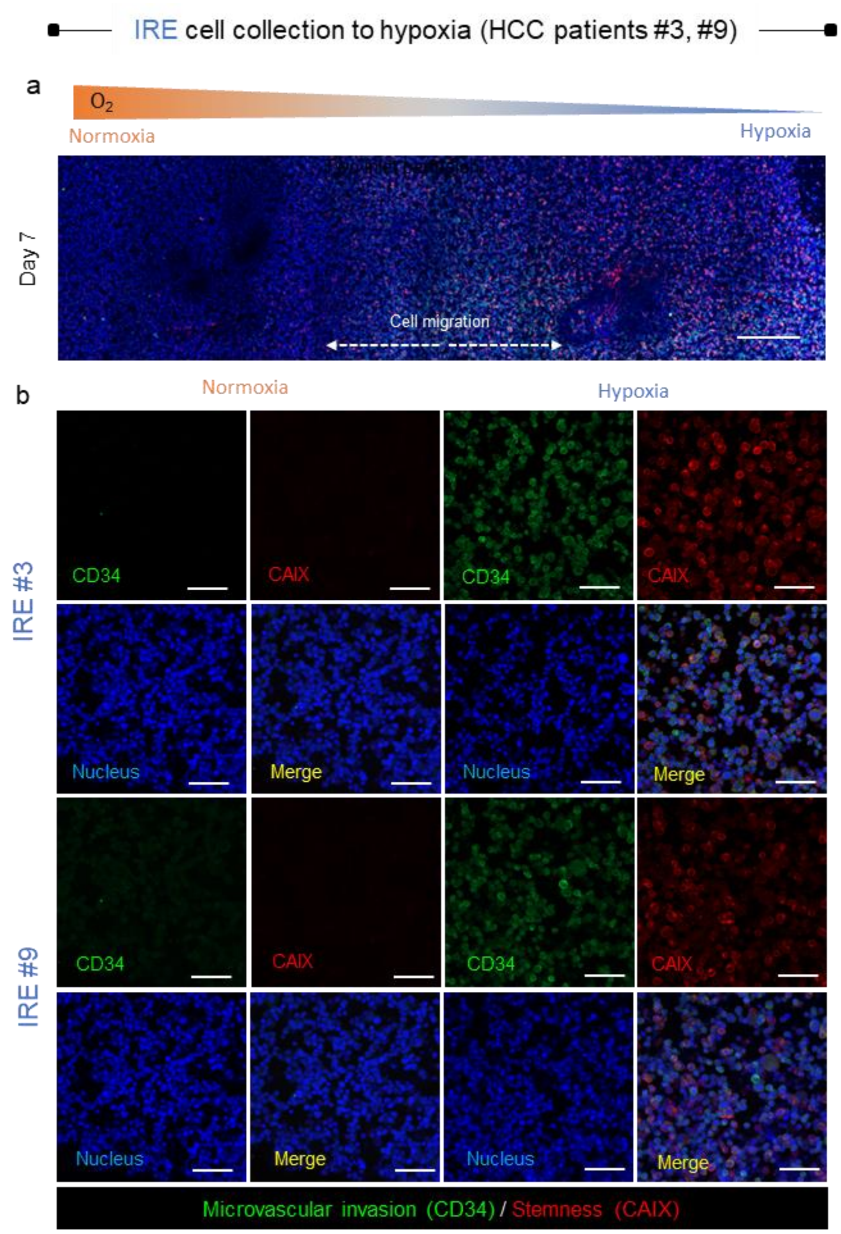

Supplementary Fig. 11 | Retrieval of IRE cells from HCC tissues using the chip. a. After 7-day culture of HCC patient tissue (#3 or #9) under dual inlet perfusion, the oxygen gradient chip enables collection of IRE cells in the hypoxic region with the incremental marker expression of microvascular invasion (CD34) and stemness (CAIX) (Scale bar = 1 mm) **b.** as confirmed by magnification (Scale bars = 100 μm).

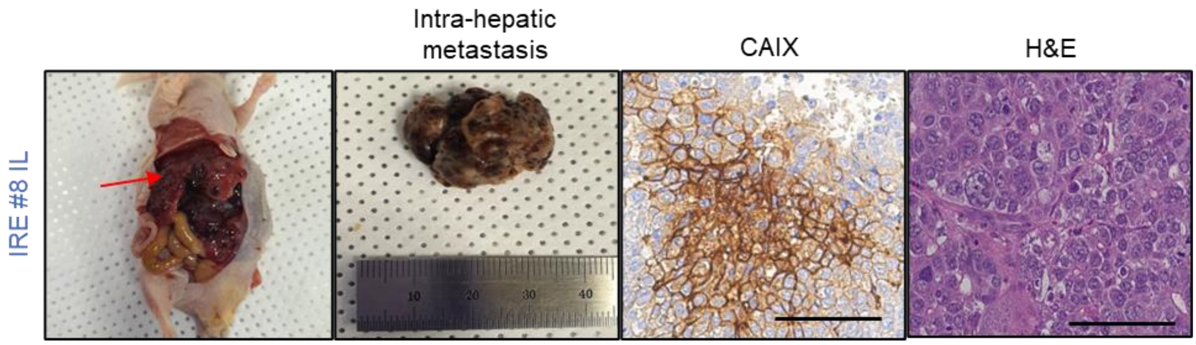

Supplementary Fig. 12 | Intra-hepatic invasion of HCC in the chip PDX model. Intra-hepatic spreading of HCC was observed upon implantation of the chip containing IRE tissue (from patient #8) into the ischemic limb of the mouse. The invasive nature of IRE HCC cells was confirmed by protein expression of CAIX in immunohistochemistry and H&E histology (Scale bars = 100μm).

Outcome	Typical<br>(n = 4)	IRE<br>(n = 8)	*P* Value
Age at diagnosis (year)	53.5±9.3	68.4±8.1	0.013
Tumor size (cm) in MRI contrast measurement	3.65±0.5	5.25±2.4	0.067
Viral infection ratio no.(%) (HBV+, HCV+)	2 (50)	6 (75)	0.828
Portal vein invasion no.(%)	1 (25)	2 (25)	1
Microvascular invasion no. (%)	2 (50)	6 (75)	0.828
Tumor necrosis (%)	2.5	16.87	0.047
AFP (ng mL^{-1}) in serum before the surgical operating	1264.15	390.26	0.173
PIVKA-II (mAU mL^{-1}) in serum before the surgical operating	98.33	10002.5	0.259
Major histological differentiation no.	Well 1<br>Moderate 2<br>Poor 1	Well 0<br>Moderate 7<br>Poor 1	0.181
Recurrence case	(0/4)	(2/8)	

Supplementary Table 1 | HCC patient information. Among the HCC patients, there were no statistically significant differences between the typical and IRE groups in terms of medical history, stage, or histological findings, except for variations in age and tumor necrosis. Over the follow-up period (median 19.8 months) in the clinic, recurrence was observed in none of the 4 typical patients (0%), while 2 recurrences (25%) were noted among the 8 IRE patients. Age at diagnosis and tumor size data are shown as mean ± SD in age and tumor size. Events, such as viral infection and recurrence ratio, were analyzed using the chi-square test. Histological differences were examined using Fisher's exact test. Tumor necrosis, AFP and PIVAK-II are shown as mean values. Significance was determined using a two-sided t-test without adjustments for multiple comparisons to the typical. Source data are provided as a Source Data file.

Gene	Forward (5′→3′)	Reverse (5′→3′)
GAPDH	TGCCATCAATGACCCCTTCAT	GGAATTTGCCATGGGTGGAAT
CAIX	GGCTACAGCTGAACTTCCGA	AAGGAGGCCTCAATCACTCG
VEGFR	AGGGAAAGGGGCAAAAACGA	ACCAACGTACACGCTCCAG
GLUT1	GAACTCTTCAGCCAGGGTCC	GAGGTCCAGTTGGAGAAGCC
ABCB1	TTGGGCTACCTATGGCTCCT	ATACACGTGGAGAAGCTGCC
ABCC2	GCATCGATCTCTCACCCTGG	AGGCCACGTGATTCTTCCAC
GSTP1	AGGACCTCCGCTGCAAATAC	GGGCAGTGCCTTCACATAGT
CD44	ACACGAAGGAAAGCAGGACC	AGCTGAGGTCACTGGGATGA
Vim	TCACCTGTGAAGTGGATGCC	CATTTCACGCATCTGGCGTT
MMP9	GGTGATTGACGACGCCTTTG	CTGTACACGCGAGTGAAGGT
AFP	CTGCTGCAGCCAAAGTGAAG	GCTGGAGTGGGCTTTTTGTG

Supplementary Table 2 | qRT-PCR primers.
