# Supplementary material for: Chip collection of hepatocellular carcinoma based on O2 heterogeneity from patient tissue
Source: Nat Commun. 2024 Jun 15;15:5117. doi: 10.1038/s41467-024-49386-8 (PMC11180182; doi:10.1038/s41467-024-49386-8)
Supplement: Supplementary file 3 — Reporting summary [file 41467_2024_49386_MOESM3_ESM.pdf]

nature portfolio

Corresponding author(s): Dai Hoon Han & Hak-Joon Sung

Last updated by author(s): May 14, 2024

Reporting Summary

Nature Portfolio wishes to improve the reproducibility of the work that we publish. This form provides structure for consistency and transparency in reporting. For further information on Nature Portfolio policies, see our Editorial Policies and the Editorial Policy Checklist.

Statistics

For all statistical analyses, confirm that the following items are present in the figure legend, table legend, main text, or Methods section.

n/a	Confirmed	
☐	☒	The exact sample size (*n*) for each experimental group/condition, given as a discrete number and unit of measurement
☐	☒	A statement on whether measurements were taken from distinct samples or whether the same sample was measured repeatedly
☐	☒	The statistical test(s) used AND whether they are one- or two-sided<br>*Only common tests should be described solely by name; describe more complex techniques in the Methods section.*
☒	☐	A description of all covariates tested
☐	☒	A description of any assumptions or corrections, such as tests of normality and adjustment for multiple comparisons
☐	☒	A full description of the statistical parameters including central tendency (e.g. means) or other basic estimates (e.g. regression coefficient) AND variation (e.g. standard deviation) or associated estimates of uncertainty (e.g. confidence intervals)
☐	☒	For null hypothesis testing, the test statistic (e.g. *F*, *t*, *r*) with confidence intervals, effect sizes, degrees of freedom and *P* value noted<br>*Give P values as exact values whenever suitable.*
☒	☐	For Bayesian analysis, information on the choice of priors and Markov chain Monte Carlo settings
☒	☐	For hierarchical and complex designs, identification of the appropriate level for tests and full reporting of outcomes
☒	☐	Estimates of effect sizes (e.g. Cohen's *d*, Pearson's *r*), indicating how they were calculated

Our web collection on statistics for biologists contains articles on many of the points above.

Software and code

Policy information about availability of computer code

Data collection	Zeiss LSM 980 was used to collect the fluorescence samples; FE-SEM confirmed the hydrogel porosity; Oxygen dissolved meter DM-1 collected the oxygen level of the chip in vitro and vivo; Ansys 2020 R1 performed simulation of oxygen diffusion; SpectraMax plate reader was used to analyze cell viability; Leica DMi-8 was used to collect the histology images; StepOnePlus v2.3 performed the qRT-PCR; ImageQuant LAS 4000 performed the western blot; Biorender.com was created schematic illustration.
Data analysis	Microsoft Excel 16.0.17531.20004, Rstudio 2023.06.1+524, Zen 3.3 blue edition, SigmaPlot 12.0, ImageQuant LAS 4000, StepOnePlus v2.3, Ansys Fluent 2020 R1, ImageJ v1.52i, GraphPad Prism 8.0.1, SoftMax®Pro Microplate .

For manuscripts utilizing custom algorithms or software that are central to the research but not yet described in published literature, software must be made available to editors and reviewers. We strongly encourage code deposition in a community repository (e.g. GitHub). See the Nature Portfolio guidelines for submitting code & software for further information.

April 2023

Data

Policy information about availability of data

All manuscripts must include a data availability statement. This statement should provide the following information, where applicable:

- Accession codes, unique identifiers, or web links for publicly available datasets
- A description of any restrictions on data availability
- For clinical datasets or third party data, please ensure that the statement adheres to our policy

Data availability
All figure and supplementary information data are available in the Figshare repository (https://doi.org/10.6084/m9.figshare.25585770.v1). The private clinical information is protected and is not available due to data privacy laws. Any additional requests for information can be directed to, and will be fulfilled by, the corresponding authors. Source data are provided with this paper.

Code availability
The heatmap.2 R code is accessible on GitHub via the following link:
https://github.com/Sewoom/RMElab/tree/Nature-communications_Liver-cancer

Research involving human participants, their data, or biological material

Policy information about studies with human participants or human data. See also policy information about sex, gender (identity/presentation), and sexual orientation and race, ethnicity and racism.

Reporting on sex and gender	N/A
Reporting on race, ethnicity, or other socially relevant groupings	N/A
Population characteristics	N/A
Recruitment	N/A
Ethics oversight	N/A

Note that full information on the approval of the study protocol must also be provided in the manuscript.

Field-specific reporting

Please select the one below that is the best fit for your research. If you are not sure, read the appropriate sections before making your selection.

☒ Life sciences ☐ Behavioural & social sciences ☐ Ecological, evolutionary & environmental sciences

For a reference copy of the document with all sections, see nature.com/documents/nr-reporting-summary-flat.pdf

Life sciences study design

All studies must disclose on these points even when the disclosure is negative.

Sample size	Sample sizes are clearly reported in the figure legends (generally n=3). The clinical participants were described in Supplementary Information 1. For the computational fluid dynamic experiments, the sample size was 1 to reduce time, and the value distribution remained unchanged. In the animal model (with n=5 independent replicates), the amount of tissue obtainable from patients was limited. Performing the maximum amount feasible in both chip and animal model (Tissue pieces 50 mg/mL were obtained from HCC patient tissues using a biopsy punch) experiments yielded n=5, which was considered a sufficient sample size for statistical analysis.
Data exclusions	No data were excluded
Replication	Replication attempts succeeded on at least two separate occasions
Randomization	The samples were assigned randomly to experimental groups
Blinding	The clinical data were collected blindly

Reporting for specific materials, systems and methods

We require information from authors about some types of materials, experimental systems and methods used in many studies. Here, indicate whether each material, system or method listed is relevant to your study. If you are not sure if a list item applies to your research, read the appropriate section before selecting a response.

Materials & experimental systems

n/a	Involved in the study
☐	☒ Antibodies
☐	☒ Eukaryotic cell lines
☒	☐ Palaeontology and archaeology
☐	☒ Animals and other organisms
☐	☒ Clinical data
☒	☐ Dual use research of concern
☒	☐ Plants

Methods

n/a	Involved in the study
☒	☐ ChIP-seq
☒	☐ Flow cytometry
☒	☐ MRI-based neuroimaging

Antibodies

Antibodies used

CAIX: NB100-417, Novus Biological LLC, rabbit monoclonal, diluted 1:1000 for immunohistochemistry, 1:500 for immunofluorescence and western blot.
CD31: NB600-562, Novus Biological LLC, mouse monoclonal, diluted 1:100 for immunohistochemistry.
K19: ab9221, Abcam, mouse monoclonal, diluted 1:1000 for immunohistochemistry.
CD34: GA63261-2, Agilent Dako, mouse monoclonal, diluted 1:50 for immunohistochemistry, 1:200 for immunofluorescence.
AFP: ab169552, Abcam, rabbit monoclonal, diluted 1:100 for immunohistochemistry.
ABCB1: sc-55510, Santa Cruz Biotechnology, mouse monoclonal, diluted 1:250 for western blot.
b-actin: sc-47778, Santa Cruz Biotechnology, mouse monoclonal, diluted 1:500 for western blot.
HRP-labeled secondary antibodies: k4003, Agilent Dako, diluted 1:5000.
F-actin: Alexa Fluor 488 phalloidin, A12379, Thermofisher, diluted 1:500.
FITC-conjugated second antibody: anti-mouse, 115-095-003, Jackson Immuno Research, diluted 1:250.
Alexa Fluor 594-conjugated second antibody: anti-rabbit, 111-585-003, Jackson Immuno Research, diluted 1:250.

Validation

All primary antibodies used were specified by the manufacturers (application, target species). Each antibody was validated by the supplier, with validation statements provided on the manufacturer's website.

Eukaryotic cell lines

Policy information about cell lines and Sex and Gender in Research

Cell line source(s)

The HCC cell line Hep3B, obtained from the Korean Cell Line Research Foundation (88064; Seoul, Republic of Korea). Derived from an 8 year old male.

Authentication

The cell line was authenticated by the STR profiling from purchasing institution. D3S1358: 15; vWA: 17; FGA: 18; Amelogenin: X; TH01: 6,7; TPOX: 9; CSF1PO: 8; D5S818: 13; D13S317: 12,14; D7S820: 8, 10.

Mycoplasma contamination

Declare that the cell line was not test mycoplasma contamination.

Commonly misidentified lines
(See ICLAC register)

No Commonly misidentified cell lines were used in the study.

Animals and other research organisms

Policy information about studies involving animals; ARRIVE guidelines recommended for reporting animal research, and Sex and Gender in Research

Laboratory animals

Male BALB/c nude mice aged six weeks, were obtained from Orient Bio (Republic of Korea)and acclimated to a pathogen-free environment with a 12-hour light/dark cycle, ambient temperature and humidity.

Wild animals

N/A

Reporting on sex

This study was conducted using male animals to avoid potential influences from hormonal fluctuations in females.

Field-collected samples

N/A

Ethics oversight

Animal experiments were conducted in accordance with the guidelines set by the Institutional Animal Care and Use Committee (IACUC) at Yonsei University College of Medicine (#2021-0176).

Note that full information on the approval of the study protocol must also be provided in the manuscript.

Clinical data

Policy information about clinical studies

All manuscripts should comply with the ICMJE guidelines for publication of clinical research and a completed CONSORT checklist must be included with all submissions.

Clinical trial registration	The clinical study was conducted following approved protocols from Yonsei University College of Medicine (IRB 4-2016-0728).
Study protocol	Diagnosed in the early stages according to the Barcelona Clinic Liver Cancer system, patients were classified into typical and irregular rim-like enhancement (IRE) HCC groups based on medical records and MRI findings. Typical HCC exhibited uniform arterial phase enhancement above the liver's overall level, while IRE HCC displayed irregular enhancement with a bright peripheral border and dim center.
Data collection	Twelve patients with HCC consented to donate tissue during surgical tumor resection at the Department of Surgery, Division of Hepato-biliary and Pancreatic Surgery, Yonsei University College of Medicine, from November 2020 to July 2023.
Outcomes	The tumor mass and MRI contrast signal intensity were analyzed using segmentation plots (Image J). Before surgery, blood samples were collected and assessed for viral infection, AFP, and PIVKA-2. Histological analysis of patients (portal vein and microvascular invasion, tumor necrosis area, differentiation) was conducted using the primary tumor mass, and follow-up (median 19.8 months) for recurrence after surgery (Supplementary Information Table 1).

Plants

Seed stocks	N/A
Novel plant genotypes	N/A
Authentication	N/A
